# Supplementary material for: Cobalt Iron Oxide (CoFe2O4) Nanoparticles Induced Toxicity in Rabbits
Source: Vet Sci. 2023 Aug 9;10(8):514. doi: 10.3390/vetsci10080514 (PMC10459303; doi:10.3390/vetsci10080514)
Supplement: Supplementary file 1 [file vetsci-10-00514-s001.zip › vetsci-2502424-supplementary.pdf]

veterinary sciences

MDPI

Table 1. Comparison of hematological profile of normal and cobalt iron oxide ($CoFe_2O_4$) nanoparticles (CIONPs) treated rabbits.

Parameters/Day	Groups/Treatments		P-Values
	A	B	
Red Blood Cells ($10^6/mm^3$)			
5	5.51±0.21	4.95±0.25	0.11
10	5.39±0.19	3.33±0.12	0.01
White Blood Cells			
5	13.35±0.41	14.2±0.15	0.12
10	13.21±0.21	17.93±0.19	0.01
Hemoglobin (gm/dl)			
5	13.5±0.16	12.6±0.31	0.13
10	13.05±0.23	9.05±0.2	0.02
Hematocrit (%)			
5	37.6±1.23	36.3±1.32	0.10
10	35.9±1.11	26.23±2.11	0.01
Neutrophils (%)			
5	22.7±1.53	31.15±1.75	0.01
10	23.3±1.13	33.91±2.31	0.01
Lymphocytes (%)			
5	24.7±1.2	23.29±0.29	0.15
10	23.3±1.1	15.4±0.19	0.01
Monocytes (%)			
5	5.83±0.43	5.11±0.12	0.10
10	6.41±0.51	4.13±0.17	0.01

Results are expressed as Mean ± S.D the values in each row are significantly different in treated rabbits as compared to untreated rabbits; (n=16; 8 each in control/untreated group A and treated group B).

Table 2. Comparison of serum biochemistry profile of normal and cobalt iron oxide ($CoFe_2O_4$) nanoparticles (CIONPs) treated rabbits.

Parameters/Day	Groups/Treatments*		P-Values
	A	B	
Alanine Aminotransferase (U/L)			
5	79.4±1.31	89.5±3.31	0.04
10	81.6±1.21	95.9±2.21	0.01
Alkaline Phosphatase (U/L)			
5	54.43±3.33	56.23±0.3	0.15
10	55.61±0.23	66.11±0.2	0.01
Albumin (mg/dL)			
5	3.69±0.16	3.62±0.13	0.10
10	3.79±0.14	2.65±0.11	0.01
Total protein (mg/dL)			
5	6.55±0.21	6.15±0.31	0.10
10	6.65±0.11	4.05±0.10	0.01
Urea (mg/dL)			
5	41.8±2.3	45.9±1.3	0.14
10	42.9±2.1	49±1.11	0.01
Creatinine (mg/dL)			
5	1.33±0.14	1.46±0.15	0.11
10	1.35±0.11	2.19±0.17	0.01
Cholesterol (mg/dL)			
5	78.1±3.41	80.5±2.4	0.13
10	79.6±4.33	93.5±2.3	0.01
Triglycerides (mg/dL)			
5	96.8±2.3	98.9±1.22	0.11
10	98.7±2.2	117.9±1.27	0.01
Glucose (mg/dL)			
5	60.5±2.2	62.1±1.13	0.12
10	61.4±2.3	78.9±2.3	0.01

*Results are expressed as Mean ± S.D the values in each row are significantly different in treated rabbits as compared to untreated rabbits; (n = 16; 8 each in control/untreated group A and treated group B).

Table 3. Antioxidant enzymes and oxidative stress biomarkers of normal and cobalt iron oxide ($CoFe_2O_4$) nanoparticles (CIONPs) treated rabbits.

Parameters/Day	Groups/Treatments*		P-Values
	A	B	
Superoxide dismutase (U/mg)			
5	28.15±1.99	26.24±1.78	0.15
10	29.55±3.1	19.11±1.4	0.01
Peroxidase (U/mg)			
5	2.83±0.17	2.71±0.13	0.14
10	2.78±0.21	1.81±0.19	0.01
Reduced glutathione(U/mg)			
5	28.9±1.56	27.3±1.07	0.10
10	30.3±1.25	22.6±1.03	0.01
Catalase (U/mg)			
5	23.5±3.1	22.6±1.03	0.01
10	24.1±1.09	14.7±2.1	0.001
Malondialdehyde concentration (U/mg)			
5	25.2±1.6	28.1±1.2	0.12
10	26.4±1.01	33.3±1.77	0.01

*Results are expressed as Mean ± S.D the values in each row are significantly different in treated rabbits as compared to untreated rabbits; (*n* = 16; 8 each in control/untreated group A and treated group B).
